# Q Fever as a Cause of Community-Acquired Pneumonia in French Guiana

**DOI:** 10.4269/ajtmh.21-0711

**Published:** 2022-08-17

**Authors:** Loïc Epelboin, Aba Mahamat, Timothée Bonifay, Magalie Demar, Philippe Abboud, Gaëlle Walter, Anne-Sophie Drogoul, Alain Berlioz-Arthaud, Mathieu Nacher, Didier Raoult, Félix Djossou, Carole Eldin

**Affiliations:** ^1^ Infectious and Tropical Diseases Department, Centre Hospitalier de Cayenne Andrée Rosemon, Cayenne, French Guiana;; ^2^ Equipe EA 3593, Ecosystèmes Amazoniens et Pathologie Tropicale, Université de la Guyane, Cayenne, Guyane française;; ^3^ Corsica Centre for Healthcare-Associated Infections Control and Prevention, Hôpital Eugénie, Ajaccio, France;; ^4^ Département Universitaire de Médecine Générale, Université des Antilles, Pointe-à-Pitre, Guadeloupe;; ^5^ Laboratoire Hospitalo-Universitaire de Parasitologie et Mycologie, Centre Hospitalier de Cayenne Andrée Rosemon, Cayenne, Guyane française;; ^6^ Institut Pasteur de la Guyane, Cayenne, French Guiana;; ^7^ Centre d’Investigation Clinique, CIC INSERM 1424, Centre Hospitalier de Cayenne, Cayenne, French Guiana;; ^8^ IHU Méditerranée Infection, Marseille, France;; ^9^ Aix Marseille University, IRD, AP-HM, SSA, VITROME, IHU-Méditerranée Infection, Marseille, France

## Abstract

In French Guiana, community-acquired pneumonia (CAP) represents over 90% of *Coxiella burnetii* acute infections. Between 2004 and 2007, we reported that *C. burnetii* was responsible for 24.4% of the 131 CAP hospitalized in Cayenne. The main objective of the present study was to determine whether the prevalence of Q fever pneumonia remained at such high levels. The secondary objectives were to identify new clinical characteristics and risk factors for *C. burnetii* pneumonia. A retrospective case-control study was conducted on patients admitted in Cayenne Hospital, between 2009 and 2012. All patients with CAP were included. The diagnosis of acute Q fever relied on titers of phase II IgG ≥ 200 and/or IgM ≥ 50 or seroconversion between two serum samples. Patients with Q fever were compared with patients with non-*C. burnetii* CAP in bivariate and multivariate analyses. During the 5-year study, 275 patients with CAP were included. The etiology of CAP was identified in 54% of the patients. *C. burnetii* represented 38.5% (106/275; 95% CI: 31.2–45.9%). In multivariate analysis, living in Cayenne area, being aged 30–60 years, C-reactive protein (CRP) > 185 mg/L, and leukocyte count < 10 G/L were independently associated with Q fever. The prevalence of Q fever among CAP increased to 38.5%. This is the highest prevalence ever reported in the world. This high prevalence justifies the systematic use of doxycycline in addition to antipneumococcal antibiotic regimens.

## INTRODUCTION

Q fever is a zoonosis caused by *Coxiella burnetii*, an intracellular bacterium, which is present worldwide.^1^ The primary infection is called acute Q fever and can manifest itself as isolated fever, hepatitis, or pneumonia.^1^ It is a public health problem in the area of Cayenne, French Guiana, a French overseas territory located in the Amazonian area of the northeastern part of South America. Although the incidence of Q fever in French Guiana is the highest in the world, by contrast, it is exceptionally described elsewhere in Latin America.^2^^–^^4^ A unique clone, the strain MST17, is responsible for most of the cases in French Guiana and shows a greater virulence than classical strains in animal models.^5^^–^^7^ While human cases are generally linked to the contact with goats or sheep worldwide, no direct evidence of carriage in cattle, goats, or sheep has been found in French Guiana. However, so far, it has been found in a few animals species, such as the three-toed sloth (*Bradypus tridactylus*), around the city of Cayenne, and more recently in capybara (*Hydrochoerus hydrochaeris*) in the context of a rural outbreak.^7^^,^^8^

Regarding clinical features, while community-acquired pneumonia (CAP) generally represents less than 50% of cases of primary *C. burnetii* infection in most series, it represents more than 90% of cases in French Guiana.^9^^–^^11^ Moreover, *C. burnetii* represents the main pathogen responsible for hospitalized CAP in Cayenne. We reported, between 2004 and 2007, that *C. burnetii* was responsible for over 24% of 131 CAP admitted in Cayenne General Hospital, the reference hospital in French Guiana.^11^ This was the highest prevalence of Q fever pneumonia ever reported in the literature, where it only ranges from 0% to 3% of CAP, depending on the region and the type of CAP (community, in hospital, intensive care unit).

The local treatment recommendations in Cayenne Hospital integrate this epidemiology and cover *C. burnetii* in the empirical treatment of CAP. The most prescribed empirical antibiotic regimen for CAP, supported by the general practitioners and the emergency department, is a combination of amoxicillin and doxycycline. Currently, the diagnosis of acute Q fever is obtained by serology, with seroconversion during the primary infection occurring between day 7 and day 21 after the onset of the symptoms, thus, in the absence of a local point of care quantitative polymerase chain reaction (qPCR) technique, other tools are useful to help choosing the antibiotic regimen. Our first publication led to a predictive score to promptly identify Q fever among hospitalized CAP.^11^ Male sex, middle age (30–60 years), headache, leukocyte count < 10 G/L, and C-reactive protein (CRP) level > 185 mg/L were thus independently associated with Q fever and were used in the predictive score.^11^ Unfortunately, exposure risk factors were not analyzed in this study. However, some exposure risks factors for Q fever in French Guiana have been described, such as living near the forest, frequently seeing bats, marsupials, or wild mammals near the house, as well as owning an air-conditioned vehicle, performing leveling work near the house, gardening, working in the building trade or public work, or house cleaning.^10^^,^^12^ Furthermore, since the previous study, *C. burnetii* DNA was detected in a dead three-toed sloth found next to a hill where an outbreak of Q fever had occurred, and carrying a three-toed sloth in arms was an independent risk factor for acute Q fever.^7^^,^^12^

The very high prevalence observed in this previous study may reflect two possible epidemiological situations: an endemic situation with persistent high incidence or an outbreak at the time of the study. To sort between these two scenarios, we completed data collection for longer stretches of time to see if the trends were the same. Thus, the main objective was to evaluate acute Q fever prevalence among CAP between 2009 and 2012. The secondary objectives were to identify clinical characteristics and risk factors for acute Q fever.

## METHODS

### Setting.

In French Guiana, the Amazonian rain forest covers over 98% of the 84,000 km^2 ^territory.^13^ The remaining part in the north is a coastal plain where 90% of the 215,000 inhabitants live, with Cayenne and surroundings (including Rémire-Montjoly and Matoury) concentrating almost 50% of the population in 2016 (URL: www.insee.fr). Its location a few degrees from the intertropical convergence zone brings a humid equatorial climate, with the alternance between wet and dry season rhythming natural cycles and human activities.

#### Patients.

We retrospectively analyzed patients with CAP admitted in the Department of Infectious and Tropical Diseases of Cayenne Hospital, French Guiana, between January 1, 2009, and December 31, 2012. Cayenne Hospital is a general hospital and the only hospital of the territory with an infectious diseases department (20 beds) and advanced microbiological diagnostic facilities for the etiologies of CAP; it is in close vicinity to the Institut Pasteur in French Guiana, which performs *C. burnetii* serology.

#### Inclusion criteria.

##### CAP criteria.

All patients who had an acute onset (≤ 7 days) of respiratory signs, which were defined as cough, chest pain, expectoration, and dyspnea, associated with at least one of the following signs: body temperature > 38°C, auscultation suggestive of pneumonia (crackles, wheezing, diminution of vesicular murmur, and vocal vibrations), and radiographic evidence of pneumonia (alveolar syndrome, interstitial syndrome, bronchial involvement, and pleural effusion) were considered to have CAP.

### Microbiological definition of an acute Q fever case.

Screening serologic tests were performed at the Institut Pasteur de la Guyane, and diagnosis was confirmed by the French National Reference Center for Q fever, Marseille, France, with an in-house indirect immunofluorescence assay, using *C. burnetii* Nine Mile strain phase I and phase II antigen, as previously described.^14^ Serodiagnosis of Q fever was performed on all patients with CAP using IgG and IgM antibodies against phase II and phase I antigens of *C. burnetii* in an indirect immunofluorescence assay. Criteria for a positive *C. burnetii* serology titers of phase II IgG ≥ 200 and/or IgM ≥ 50. Criteria for the diagnosis of acute Q fever were the following: seroconversion,^15^ identified between two serum samples (one from the acute phase and one from the convalescent phase) OR one positive serology (phases I and II) associated with no other identified etiology and diagnosis of Q fever by the clinician in charge of the patients, treated by doxycycline.

#### CAP microbiological screening.

Patient with CAP of unknown or other etiology were defined as controls. While all patients were routinely serologically tested for *C. burnetii*, due to the known importance of this infectious pathogen in French Guiana, microbiological diagnosis of other respiratory infectious agents was performed at the discretion of the attending physician. Diagnostic tests for other pneumonia pathogens were performed. Serologic tests were used to detect *Mycoplasma pneumoniae, Chlamydia pneumoniae, Chlamydia psittaci, Legionella pneumophila, Toxoplasma gondii* (its Amazonian strains causing severe pneumonia), and *Bordetella pertussis*. The identification of *L. pneumophila* and *Streptococcus pneumoniae* were based on the detection of specific antigens in urine samples by an immunochromatographic assay and blood cultures. Serologic tests and qPCR were performed to detect arbovirosis, measles, and leptospirosis. If pulmonary tuberculosis was suspected, specific direct examination and cultures of sputum smears were performed.

#### Ethics statement.

Patient’s medical records were retrospectively reviewed. All data collected were anonymized using standardized forms according to the procedures of the Commission Nationale de l’Informatique et des Libertés (the French information protection commission). The study was approved by the local ethics committee as this research was consistent with the French MR004 reference methodology and fell within the framework of so-called “internal” research, that is, the data were collected as part of the individual follow-up of patients, by the staff ensuring this follow-up and for their exclusive use. In accordance with the European regulation on data protection, the study was registered in the hospital’s registry of processing activities by the person in charge of data protection, and collective information was made available to patients by means of a poster in the department, allowing them to express their refusal to participate, if necessary.

#### Data collection and analysis.

The acute Q fever and CAP cases were identified retrospectively using the French PMSI (Programme Médicalisé des Systèmes d’Information) through the extraction of all patients receiving diagnostic codes of the CIM-10 for pneumonia: J12, J13, J15, J16, J17, J18, and the A78 code for Q fever.

A standardized form was completed retrospectively for data collection, including socio-epidemiologic data, clinical symptoms of the first clinical examination, and radiographic and biological results. Risk factors were also included in the data collection. The variable was considered positive if present in the file and negative if absent. All data were recorded retrospectively with Microsoft Excel. The continuous variables of interest were categorized following the laboratory cut-off values or values in the medical literature. They generally were dichotomized because of the sample size.

### Determination of risk factors.

A case-control study was performed to identify factors associated with Q fever among CAP, comparing patients with Q fever CAP (cases), to patients with CAP of other etiology or unknown etiology (controls). Statistical analysis of data was performed using the SPSS Software (IBM), version 22.0. Categorical variables were described using frequencies and percentages and compared using Fisher’s exact test. Continuous variables were described using means ± SDs or medians with interquartile ranges; they were compared using the Mann–Whitney test. Logistic regression analysis was performed. Potential variables associated with Q-fever CAP occurrence were selected using bivariate logistic regression. The variables retained after the bivariate analysis (*P* < 0.20) were introduced in a multivariate logistic regression model with a backward step-by-step selection procedure. The significance level *p* was fixed at 0.05. The estimated adjusted odd ratios (OR) were presented with their 95% CI (95% CI).

### Review of the literature about acute Q fever prevalence.

After identification of a high prevalence of Q fever CAP in our study, we performed a search in Medline with the following keywords: [Acute Q fever] [*Coxiella burnetii*] [prevalence] [Hospital] [Pneumonia] from January 01, 1980, to December 31, 2021.

## RESULTS

### Baseline characteristics of the study population.

During the 5-year study period, 275 patients with CAP were included. Among this population, 66% were male and the mean age was 46.8 years (range: 15.9–97.3, SD = 16.4) (Table 1). Forty-one percent of patients were natives of French Guiana and 23% were born in mainland France. The majority of patients lived in the Cayenne area (85.8%). Overall, 36% smoked tobacco. In this cohort, the etiology of CAP was not identified for 46.2% of patients. Among patients with a known etiological agent, the first pathogen was *C. burnetii*, representing 38.5% (106/275; 95% CI = 31.2–45.9%) of all patients and 71.6% (106/148; 95% CI = 58.0–85.3%) of those who had a microbiological identification (Table 2). Twenty-nine patients were diagnosed based on a seroconversion, 61 patients were diagnosed based on elevated phase II IgG and IgM titers (both positive on two serum samples) without seroconversion, 13 patients were diagnosed based on a one-off serology with both positive phase II IgG and IgM, and 3 patients were diagnosed based on a one-off positive phase II IgM. The prevalence of Q fever among acute CAP fluctuated from year to year with no clear trend, ranging from 29.2% (2010) to 68.8% (2009) (Table 3). The second most frequent bacterium was *Mycoplasma pneumoniae* in 21 patients (7.6%), followed by *Streptococcus pneumoniae* (1.8%) and the Amazonian strain of *Toxoplasma gondii*, so-called Amazonian toxoplasmosis, for 4 patients (1.5%). Most patients presenting with CAP in Cayenne Hospital had fever (90%) and cough (66%) and radiological abnormalities were present in 83% of cases (Table 1). Digestive manifestations (abdominal pain, diarrhea, and vomiting) were present in 53% of cases and eye, nose, and throat symptoms or signs were reported in 47% of patients.

**Table 1:** Baseline characteristics and comparison of sociodemographical features between cases and controls

Characteristics	Variables	Q fever CAP (*N* = 106) *N* (%)	Non-Q fever CAP (*N* = 169) *N* (%)	All CAP (*N* = 275) *N* (%)	OR (95% CI)	p*
Age	Mean (±SD) (years)	47.3 (±13.4)	46.4 (±18.1)	46.8 (±16.4)	–	NS
	Age 30–60 years vs. others	76 (71.7)	94 (55.6)	179 (65)	2.02 (1.17–3.53)	0.008
Gender	Male sex	70 (66)	96 (56)	166 (60)	1.48 (0.87–2.53)	0.13
	Sex ratio M/F	1.9	1.3	1.5	–	–
Country/region of birth	Mainland France	32/102 (30.2)	32/164 (19.5)	64 (23)	1.89 (1.02–3.47)	0.03
	French Guiana	48/102 (47.1)	65/164 (39.6)	113 (41)	1.36 (0.80–2.30)	0.23
	Brazil	7/102 (6.9)	24/164 (14.6)	31 (11.2)	0.43 (0.18–1.02)	0.05
	Other	15/102 (14.7)	43/164 (26.2)	58 (21)	0.49 (0.26–0.92)	0.03
Place of residence	Cayenne	41/102 (40.2)	73/166 (44.0)	114 (42.5)	0.86 (0.50–1.45)	0.54
	Rémire-Montjoly	30/102 (29.4)	30/166 (18.1)	60/268 (22.4)	1.89 (1.06–3.37)	0.03
	Matoury	26/102 (25.5)	30/166 (18.1)	56/268 (20.9)	1.55 (0.82–2.93)	0.15
	Cayenne area¤	100/106 (94.3)	140/169 (82.8%)	240/275 (85.8)	4.8 (1.8–16.2)	< 0.001
Comorbidities	Sickle cell disease	0/84 (0)	13/155 (8.4)	13 (5)	–	0.006
	Tobacco smoking	52/100 (52)	48/168 (28.6)	100 (36.3)	2.71 (1.57–4.69)	< 0.001
	Cannabis smoking	5 (4.7)	5 (3)	10 (3.6)	1.62 (0.36–7.23)	0.45
	Crack smoking	1/101 (1)	7/168 (4)	8 (3)	0.23 (0.01–1.84)	0.14
	Alcohol	54/100 (54.0)	65/168 (38.7)	119 (43.2)	1.86 (1.09–3.17)	0.015
	PLHIV	1/90 (1.1)	6/149 (3.9)	7 (2)	0.28 (0.01–2.34)	0.21
Environmental exposure	Leveling work near the house	27/88 (25.4)	20/168 (11.8)	47 (17.4)	4.23 (2.13–8.45)	< 0.001
	Gardening	23 (26.1)	19 (11.3)	42 (15.2)	4.70 (2.46–9.12)	< 0.001

CAP = community-acquired pneumonia; NS = not significant; PLHIV = people living with human immunodeficiency virus; OR = odds ratio. Cayenne area = Cayenne + Rémire-Montjoly + Matoury.

* *P* value obtained by bivariate logistic regression.

**Table 2:** Etiology of community-acquired pneumonias admitted in the department of infectious and tropical diseases, Cayenne Hospital, French Guiana, 2009–2012

Microbiological agent associated with CAP	*N *(%)
*Coxiella burnetii*	106 (38.5)
*Mycoplasma pneumoniae*	21 (7.6)
*Streptococcus pneumoniae* *	5 (1.8)
*Toxoplasma gondii*†	4 (1.5)
Dengue virus	2 (0.7)
*Leptospira* sp.	2 (0.7)
*Bordetella pertussis*	2 (0.4)
Tuberculosis	1 (0.4)
*Chlamydia pneumoniae*	1 (0.4)
*Escherichia coli*	1 (0.4)
Epstein-Barr virus	1 (0.4)
*Haemophilus influenzae*	1 (0.4)
Measles	1 (0.4)
Unknown	127 (46.2)
Total	275 (100)

CAP = community-acquired pneumonia.

* Two diagnosis with blood cultures, two with *S. pneumoniae* urinary antigen, and one with both.

† In French Guiana, infection with a virulent strain of *Toxoplasma gondii* in immunocompetent patients is frequent and so-called Amazonian toxoplasmosis.

**Table 3:** Prevalence of Q fever among pneumonias admitted in the Cayenne Hospital 2009–2012 according to the year

Year	Not Q fever	Q fever	Total	Q fever prevalence (%)	95% CI (%)
2009	5	11	16	68.8	28.1–109.4
2010	97	40	137	29.2	20.1–38.2
2011	46	29	75	38.7	24.6–52.7
2012	21	26	47	55.3	34.0–76.6
Total	169	106	275	38.5	31.2–45.9

#### Variables associated with Q fever versus other etiology among CAP.

In bivariate analysis, sociodemographic characteristics of patients associated with *C. burnetii* CAP versus other etiologies were age between 30 and 60 (*P* < 0.001), being born in mainland France (*P* = 0.03), living in Cayenne and its suburbs (*P* < 0.001), reporting tobacco and frequent alcohol consumption (*P* < 0.001 and *P* = 0.01, respectively), and the notion of leveling work near the house and gardening activities (*P* < 0.001) (Table 1).

Regarding clinical features, the presence of temperature > 38.5°C, headaches, chills, muscle pain, and abnormal auscultation were associated with acute Q fever CAP in bivariate analysis (Tables 4 and 5). In addition, acute Q fever CAP were significantly associated with the absence of cough and eyes nose throat (ENT) symptoms in bivariate analysis. The analysis of biological data found that white blood cell count (WBC) inferior to 10 G/L, polymorph nuclear (PMN) count inferior to 7.7 G/L, CRP > 185 mg/L, and serum glutamo pyruvate transaminase (TGP) and/or serum glutamo oxaloacetic transaminase (TGO) greater than 1.5 times the normal range were significantly associated with Q fever CAP in bivariate analysis (Tables 1 and 4).

**Table 4:** Comparison of clinical, biological, and radiological features of Q fever patients and controls, bivariate analysis

	Clinical features	Q fever CAP (*N* = 106) *N* (%)	Non-Q fever CAP (*N* = 169) *N* (%)	All CAP (*N* = 275) *N* (%)	OR (95% CI)	*p*
Physical examination	Temperature > 38.2	56/95 (59.0)	63/155 (40.7)	119/250 (47.6)	2.1 (1.2–3.7)	0.005
	Mean temperature (±SD) (°C)	38.4 (±1.2)	38.0 (±1.3)	38.1 (±1.3)	–	0.01
	Headache	64 (60.4)	65/168 (38.7)	129 (47.1)	2.4 (1.4–4.1)	< 0.001
	Chills	51 (48.1)	59/168 (35.1)	110 (40.1)	1.7 (1.1–2.8)	0.03
	Muscle pain	55 (51.9)	60/168 (35.7)	115 (42.0)	1.9 (1.2–3.2)	0.008
	Cough	62 (58.5)	119/168 (70.8)	181 (66.1)	0.6 (0.4–0.9)	0.04
	Chest pain	24 (22.6)	53/168 (31.5)	77 (28.1)	0.6 (0.4–1.1)	0.11
	Dyspnea	14 (13.2)	37/168 (22.0)	51 (18.6)	0.5 (0.3–1.1)	0.07
	Ear nose and throat symptoms	8 (7.6)	39/165 (23.6)	47 (17.6)	0.3 (0.1–0.6)	< 0.001
	Abdominal pain	16 (15.1)	17/164 (10.4)	33 (12.2)	1.5 (0.8–3.2)	0.25
	Diarrhea	14/104 (13.5)	33/162 (20.4)	47 (17.7)	0.6 (0.3–1.2)	0.15
	Vomiting	28 (26.4)	38/163 (23.3)	66 (24.5)	1.2 (0.7–2.1)	0.56
	Abnormal pulmonary auscultation	50/95 (52.6)	129/165 (78.2)	179/260 (68.8)	0.3 (0.2–0.6)	< 0.001
Chest X-rays	Abnormal radiography	63/83 (75.9)	145/168 (86.3)	208/251 (82.9)	0.5 (0.2–1.0)	0.05
Laboratory results						
	Leukocytes < 10 G/L	84/101 (83.2)	91/163 (55.8)		4.2 (2.1–7.6)	< 0.001
	Neutrophils < 7.7 G/L	82/97 (84.5)	100/162 (61.7)		3.4 (1.7–6.9)	< 0.001
	Lymphocytes < 1 G/L	22/95 (23.2)	33/160 (20.6)		1.2 (0.6–2.2)	0.64
	CRP > 185 mg/L	56/98 (57.1)	57/167 (34.1)		2.6 (1.5–4.4)	< 0.001
	Platelet count < 150 G/L	20/96 (20.8)	23/161 (14.3)		1.6 (0.8–3.2)	0.17
	SGPT and/or SGOT > 1.5 N	48/96 (50.0)	54/154 (35.1)		1.9 (1.1–3.2)	0.02
	Creatinine > 126 µM)	5/88 (5.7)	14/158 (8.9)		0.6 (0.2–1.9)	0.37

CRP = C-reactive protein; OR = odds ratio; SGOT = serum glutamooxaloacetate transférase; SGPT = serum glutamo pyruvate transférase.

**Table 5:** Comparison in multivariate analysis of clinical, biological, and radiological features of Q fever patients and controls supported between 2009 and 2012 in Cayenne General Hospital, and comparison to the results of the first study 2007–2004

Study period	2009–2012		2004–2007	
Multivariate analysis	aOR	95% CI	aOR 95% CI	95% CI
To be living in Cayenne area	3.6	1.3–10.0	–	–
Male gender	–	–	4.8	1.3–17.9
Age 30–60 years	2.1	1.2–3.8	5.0	1.5–16.8
Headache	–	–	4.4	1.6–12.4
CRP > 185 mg/L	3.1	1.7–5.5	4.1	1.4–11.8
Leukocytes < 10 G/L	4.54	2.4–8.7	7.3	1.9–27.4

aOR = adjusted odds ratio.

In the multivariate analysis, living in Cayenne area, being aged between 30 and 60 years old, having a CRP levels > 187 mg/L, and having WBC counts <10 G/L were independently associated with Q fever (Table 4).

## DISCUSSION

### The highest prevalence in the world ever described among CAP.

While our previous study reported that almost 25% of the CAP hospitalized in the hospital of Cayenne were due to *C. burnetii* infection, the present study, spanning the 5 years after the initial study, now found that 38.5% of CAP were Q fever cases; hence, a prolonged period with a very high proportion of Q fever cases, which is more in favor of high endemicity than a punctual outbreak.^11^ This is the highest proportion of Q fever among pneumonias ever reported in the world, even in epidemics (Table 6). Most of the international series show a prevalence of Q fever among pneumonia from 0% to 3%, the higher proportions being linked to epidemics.^16^^–^^19^ Furthermore, French Guiana is located in Latin America where Q fever is hardly reported, especially in pneumonias.^4^^,^^20^^–^^24^ The increase of the prevalence between the 2004–2007 and 2009–2012 period maybe due to a greater awareness of physicians after the first study, and a greater use of the serodiagnostic test. Indeed, in our previous study, the second sample at day 14–21 after the beginning of the symptoms was not systematically performed, while in the second study period, the seroconversion was more systematically screened with an early and a late sample. Thus, the result of 38.5% of *C. burnetii* among acute CAP is probably closer to the real prevalence than in our first study.

**Table 6:** Prevalence of *Coxiella burnetii* among acute community-acquired pneumonia

Continent	Region of study (city)	Name of first author	Year of publication	Study period	Patients and study category	Sample size	Prevalence of Q fever
Europe	United Kingdom (Nottingham)	Woodhead^29^	1987	NK	Community	0/236	0%
	United Kingdom (5 studies)	BTS^16^	1981–1982–19841987–2001	NK	In hospital	13/1137	1.2%
	United Kingdom (4 studies)	BTS^16^	1985–19901992–1997	NK	ICU	0/185	0%
	Western Europe (6 studies: Italy, Norway, Spain, Sweden)	BTS^16^	1986–1991–19921993– 1995–2000	NK	Community	5/654	0.8%
	Western Europe (23 studies)	BTS^16^	2001	NK	In hospital	36/2026	0.6%
	Western Europe (10 studies)	BTS^16^	2001	NK	ICU	8/1148	0.7%
	France	SPILF^17^	2006*		Pneumonias	UK	0%
	The Netherlands (Bernhoven)	Limonard^30^	2012	2007	In hospital	21/95	22%
	The Netherlands (Nieuwegein)	Meijvis^31^	2011	2007–2010	In hospital	27/304	8.9%
	The Netherlands (Nieuwegein)	Endeman^32^	2008	2004–2006	In hospital	1/201	0.5%
	The Netherlands (Alkmaar)	Snijders^33^	2010	2005–2008	In hospital	0/213	0%
	The Netherlands (Alkmaar)	Van der Eerden^34^	2005	1998–2000	In hospital	0/262	0%
	The Netherlands (Tilburg)	Huijskens^35^	2016	2008–2009	Emergency room	50/404	12.3%
	The Netherland (Tilburg)	Huijskens^36^	2013	2008–2009	Emergency room	27/408	6.8%
	The Netherlands (Nieuwegein and Ede)	Spoorenberg^37^	2014	2004–2010	In hospital	28/505	5.5%
	Switzerland (Lausanne)	Bochud^38^	2001	1998–2000	Outpatients	4/170	2.4%
	Spain (Barcelona)	Sopena^39^	1999	1994–1996	Emergency room	4/392	1%
	Spain (Barcelona)	Ruiz^40^	1999	1996–1997	In hospital	6/395	1.5%
	Spain(13 Spanish hospitals)	Sahuquillo-Arce JM^41^	2016	2005–2007	In hospital	51/4304	1.2%
	Spain (Navarra)	Carrillo de Albornoz^42^	1991	1988	Emergency room	18/225	8%
	Spain (Bilbao)	Obradillo^18^	1989	1982–1986	Community	31/164	18.8%
	Greece (Northern)	Alexiou-Daniil^43^	1990	1990	Atypical pneumonias	170/3686	4.7%
	Italy (San Patrignano)	Boschini^44^	1996	1991–1994	Community in IDUs	3/210	1.4%
	Germany	Schack^19^	2014	2005	In hospital	9/255	3.5%
	Israel (Beer-Sheva)	Lieberman^26^	1995	1991–1992	In hospital	20/346	5.8%
	Israel (Afula)	Shibli^45^	2010	2006–2007	In hospital	8/126	6.3%
North America	Canada (Halifax)	Marrie^46^	1996	199–1994	Community	4/149	2.7%
	Canada (Halifax)	Marrie^47^	1989	1981–1987	In hospital	22/588	3.7%
	USA (Pittsburg)	Fang^48^	1990	1986–1987	In hospital	0/359	0%
	USA (5 hospitals Chicago and Nashville)	Jain^49^	2015	2010–2012	In hospital	0/2259	0%
	USA (Maryland)	Mundy^50^	1995	1990–1991	In hospital	0/385	0%
Latin America	French Guiana (Cayenne)	Epelboin^11^	2012	2004–2007	In hospital	32/131	24.4%
	French Guiana (Cayenne)	Epelboin (this study)	2020	2008–2012	In hospital	106/275	38.0%
	Chile (Santiago)	Diaz^20^	2007	2003–2005	In hospital	0/176	0%
	Argentina (Buenos Aires)	Luna^51^	2011	1997–1998	Emergency room and community	1/346	0.3%
	Brazil (Montenegro)	Bahlis^52^	2018	2014–2015	In hospital	0/459	0%
	Brazil 5 countries of South America (Mexico, Chile, Argentina, Uruguay, Brazil)	Jardim^53^	2003	1997–1998	In hospital	0/84	0%
	Guatemala (Cuilapa and Quetzaltenango)	Contreras^54^	2015	2008–2012	In hospital	0/188	0%
Asia	Singapore	Chiang^55^	2007	3 years (NK)	In hospital children	0/1702	0%
	India	Panjwani^56^	2015	To 2015	Littérature review		0%
	India (Bangalore)	Raj Gangoliya^57^	2016	2014–2015	Atypical pneumonia	2/77	2.5%
	Taiwan	Lai^58^	2014	2012–2013	In hospital	0/166	0%
	China (Nanjing)	Chen^59^	2014	2011–2013	In hospital children	1/1204	0.08%
	China (Guangzhou)	Lin^60^	2015	2011–2012	In hospital children	26/20160	0.13%
	China (Wuhan)	Liu^61^	2015	2010–2012	In hospital children	21/39756	0.05%
	China (Hubei)	Wu^62^	2014	2010–2012	In hospital children	42/10435	0.4%
	Japan (Okayama)	Okimoto^63^	2004	2001–2003	In hospital	4/284	1.4%
Oceania	New Zealand (Waikato)	Karalus^64^	1991	1988	In hospital	0/92	0%
	New Zealand (Christchurch)	Neill^65^	1996	1992–1993	In hospital	0/255	0%
	Australia (Adelaide)	Lim^66^	1989		In hospital	0/106	0%
Africa	Cameroon (Yaounde)	Koulla-Shiro^67^^,^^68^	1996 and 1997	1991–1993	In hospital	6/91	6.5%
	South Africa (Cape Town)	Maartens^69^	1994	1987–1988	In hospital	0/92	0%

COPD = chronic obstructive pulmonary disease; ICU = intensive care unit; IDUs = intravenous drug users; NK = not known; UK = United Kingdom; USA = United States of America; BTS = British Thoracic Society; SPILF = Société de Pathologie Infectieuse de Langue Française.

* Data from the different recommendations: Société de Pathologie Infectieuse de Langue Française 1991 updated 2000; Agence française de sécurité sanitaire des produits de santé 2005.

Recent studies have demonstrated that the unique clone MST17 found in French Guiana is more virulent *in silico*, in vitro, and in vivo than the reference strains Nine Mile and the Z3055 strain, isolated from an ewe in Germany and phylogenetically very close to the strain that caused an important outbreak in the Netherlands,^5^^,^^6^^,^^25^ potentially explaining this high prevalence, at least in part. Also, the spread of the reservoir still remains unclear. The first reservoir identified was the three-toed sloth (*Bradypus tridactylus*), in the Cayenne area. However, the recent detection of *C. burnetii* DNA in capybara (*Hydrochoerus hydrochaeris*)^7^^,^^8^ in the context of a rural outbreak far from the island of Cayenne, suggests that several animals living in the Amazonian rainforest could be involved in the dissemination of *C. burnetii* infection. The first reported case of acute Q fever in Cayenne in 1998 was observed in a slaughterhouse worker, so that one could hypothesize that the bacterium may have been transmitted from its classical reservoirs to new hosts in a territory of high wild biodiversity. Moreover, these wild animals are also present in the rest of French Guiana and in all Amazonian countries. Further studies are required to understand this very high prevalence, especially in the Latin-American context, and to clarify the patterns of the wild/domestic reservoir and population features.

#### Very specific features associated with C. burnetii infection.

In the present study, we found the same features that had been described before, which are confirmed to be quite specific. *Coxiella burnetii* pneumonia is more frequently found in men, aged between 30 and 60 years old. This is often observed in zoonoses linked to professional or leisure outdoor activities and is quite close to risk groups found elsewhere.^10^^,^^26^ However, the two new very specific characteristics in French Guiana are that people born in mainland France, which represent less than 10% of the global population of the territory, were over-represented here, and had a 2-fold increase of acute Q fever than patients of other origins; however, this was no longer significant in multivariate analysis. Finally, living in Cayenne and its surroundings remained associated with the risk to get acute Q fever compared with people living elsewhere. A hot spot of Q fever was reported in the town of Rémire-Montjoly, located at the immediate East of Cayenne, without clear explanations. The city of Rémire-Montjoly is characterized by housing estates separated by hills of the Amazonian rainforest, like Bourda and Rorota hills. Previous work based on geolocation of cases had shown that these two areas were marked by the very high incidence of Q fever.^10^ These forest hills are home to three-toed sloths, one of the putative reservoirs of the disease. This difference between “Caucasian people,” sometimes including people of various color of skin but born in mainland France, compared with people born in the region may be explained by a higher immunity in the native population. Further seroprevalence studies in French Guiana could be interesting to further investigate this phenomenon. Also, one could hypothesize that people from mainland France would be more likely to consult in the hospital than those from other cultural groups, but this is not confirmed by our results showing that natives of French Guiana represented 40% of hospitalized CAP versus 23% of people born in mainland France (Table 3).

Finally, we found environmental exposure risk factors, such as gardening or work involving soil near homes. These activities had already been described earlier, and may be associated with an aerosolization of the dust, which may contain particles of the bacterium.^8^^,^^10^^,^^12^ Unfortunately, this study was performed before the discovery of the possible carriage of *C. burnetii* by wild fauna, such as three-tooth sloth and capybara, so the survey could not question the contact of the patients with these animals.^7^^,^^8^

### Practical consequences of these results.

Although the first publication suggested that doxycycline should be used only in patients with a high probability of Q fever, the results of the present study are in favor of systematic use of an anti-*C. burnetii* antibiotic regimen when facing a clinical and radiological picture of CAP due to its exceptionally high prevalence. Macrolides, which are the second-choice antibiotic regimen in addition to the antipneumococcal treatment, should not be used as the Guianese strain MST 17 seems to be resistant to this antibiotic family.^27^^,^^28^

### Limitations of the study.

This study is retrospective, and consequently suffers from several biases and unknown confounders. First, diagnostic criteria for Q fever CAP were heterogeneous, since seroconversion was not systematically identified due to the absence of follow-up for some patients. Second, etiologic investigations were left to the appreciation of the clinicians in charge of the patients, so that not all patients were tested for all the pathogens found in the study. However, we think that the high prevalence of Q fever in these real-life conditions is an important signal. Further prospective cohort studies with more stringent criteria are needed to confirm these data and to increase our knowledge about the long-term consequences of acute Q fever in French Guiana.

In conclusion, we observed a persistently high proportion of Q fever among adults with CAP in Cayenne Hospital. The relatively long time span seems more in favor of high endemicity than the punctual outbreak. This stable fact justifies the adaptation of treatment protocols for CAP, which should systematically cover for Q fever.
